# Development and evaluation of an interactive case-based training tool for timely on-farm euthanasia decision-making in swine

**DOI:** 10.1186/s40813-025-00483-0

**Published:** 2026-01-08

**Authors:** Laya Kannan Silva Alves, Cecília Archangelo Ferreira de Melo, Monique Danielle Pairis-Garcia, Andréia Gonçalves Arruda, Cesar Augusto Pospissil Garbossa

**Affiliations:** 1 Laboratory of Swine Research, Department of Animal Nutrition and Production, School of Veterinary Medicine and Animal Science, University of São Paulo, Pirassununga, São Paulo Brazil; 2 Global Production Animal Welfare Laboratory, Department of Population Health and Pathobiology, College of Veterinary Medicine, North Carolina State University, Raleigh, NC USA; 3 Department of Veterinary Preventive Medicine, College of Veterinary Medicine, The Ohio State University, Columbus, OH USA

**Keywords:** Swine welfare, Euthanasia decision-making, Veterinary education, Interactive learning, Knowledge improvement

## Abstract

Timely and humane euthanasia is essential to safeguard animal welfare on pig farms, yet studies in Brazil and other major pig-producing countries reveal persistent uncertainty and inconsistency in on-farm decision-making. This study aimed to develop and evaluate an interactive, case-based training tool to assess its impact on knowledge, confidence, and decision-making related to on-farm swine euthanasia among veterinary and animal science students. Outcomes included pre- and post-training self-assessed knowledge and confidence, accuracy of decisions in interactive case-based scenarios, and participant feedback regarding usability, realism, and perceived value of the tool. A total of 47 students participated in the study. They completed pre-and post-training Likert-scale surveys assessing self-perceived competencies related to swine euthanasia, followed by five clinical case scenarios requiring real-world decision. Participants’ demographics, education, and experience were collected and their feedback on the training tool was surveyed. Statistical analysis included Wilcoxon signed-rank tests for pre-post comparisons and multivariable logistic regression models to explore associations between participants’ characteristics and improvements in individual survey items. All eight self-assessed competencies showed statistically significant improvement after training (*p* < 0.05). Students demonstrated an average of 89.1% correct responses in interactive case-based decisions. Multivariable logistic models identified specific characteristics associated with greater improvement in confidence and knowledge, including gender, ethnicity, and prior swine experience. Participant feedback was overwhelmingly positive, with over 90% agreeing the tool was realistic, relatable, and worthwhile. These findings show the tool effectively strengthens euthanasia-related knowledge and confidence, supporting its adoption in veterinary and animal-science education to promote timely, ethical decision-making in Brazilian swine systems.

**Supplementary Information:**

The online version contains supplementary material available at 10.1186/s40813-025-00483-0.

## Introduction

Euthanasia is an ethically justified and necessary act on swine farms that must be performed in a timely manner when an animal is suffering [1, 2]. In swine production, timely euthanasia – defined as the humane decision and act of ending the life of a pig showing irreversible suffering or poor prognosis before prolonged pain or distress occurs [3, 4] – is a critical component of animal welfare, recognized within the Five Domains Model under Domain 3: Health. Pigs that exhibit clinical signs of disease, severe injury or functional impairment may experience several negative affective states, including, but not limited to, pain, weakness, and exhaustion [5]. These animals are candidates for euthanasia when no effective treatments are available or when the prospects for recovery are poor or limited [4]. When applied appropriately, euthanasia is considered a humane practice that prevents unnecessary suffering and distress and should be readily available as a management tool on the farm [3].

In Brazil, euthanasia is classified as a clinical procedure authorized solely for veterinarians [6]], with formal guidance provided by the Federal Veterinary Medical Council [1] and the Ministry of Agriculture and Livestock [4]. Veterinarians working on farms are responsible for not only performing euthanasia but also making the decision on when euthanasia is warranted. In systems where veterinarians are not present on-site daily, they must train farm caretakers to assume the responsibility to euthanize under the direct guidance and protocols established by the veterinarian [7, 8].

Although euthanasia occurs routinely on Brazilian swine farms, there are limited resources and training opportunities available to support veterinarians in the development and consistent implementation of euthanasia procedures and protocols. Despite the critical role the veterinarian plays in establishing humane and timely euthanasia in animal production systems, access to both formal and informal training remains limited, particularly in regard to practical available resources that can be used to train personnel across multiple farms [8–10]. In academic settings, most veterinary programs offer some theoretical training on euthanasia, coursework and lectures are often limited to companion animals, with minimal or no practical experience on performing euthanasia in livestock species [9]. As a result, graduating veterinarians may feel unprepared to make critical euthanasia decisions or lack the confidence to effectively train and supervise farm personnel responsible for carrying out euthanasia [11, 12]. Without adequate training at the university level, inconsistencies in euthanasia practices and reduced confidence among early career professionals in veterinary medicine and animal science may occur [13].

In response to these challenges, this study was structured in two complementary stages: first, to develop and refine an interactive, case-based training tool focused on swine euthanasia; and second, to evaluate its educational effectiveness among veterinary medicine and animal science students. These students represent future professionals who are expected to educate and supervise farm personnel and develop and implement standard operating procedures pertinent to euthanasia. By targeting the ability to identify compromised animals and act promptly, the training tool aims to support improvements in animal welfare that are consistent with the five domains model. The central hypothesis of our study was that the use of this training tool would improve self-assessed technical knowledge and decision-making confidence related to euthanasia, thereby contributing to the adoption of improved animal welfare practices on Brazilian swine farms.

## Material and methods

The study was conducted according to the guidelines of the Institutional Research Ethics Committee for Human Subjects (CEPH) of the Faculty of Animal Science and Food Engineering (FZEA) at the University of São Paulo (USP; #5.674.052).

### Development of the multimedia training tool

The multimedia training tool developed in this study consisted of two main components: a theoretical module and interactive case-based modules. The tool was designed following the euthanasia training framework proposed by Mullins et al. [12], originally developed for pig euthanasia and later applied and evaluated by Campler et al. [14] in swine and by Merenda et al. [15] in dairy cattle. The framework was adopted to guide the structure and educational objectives of the training tool, with the goal of improving knowledge, critical thinking, and confidence in euthanasia decision-making under realistic production conditions.

**Fig. 1:**
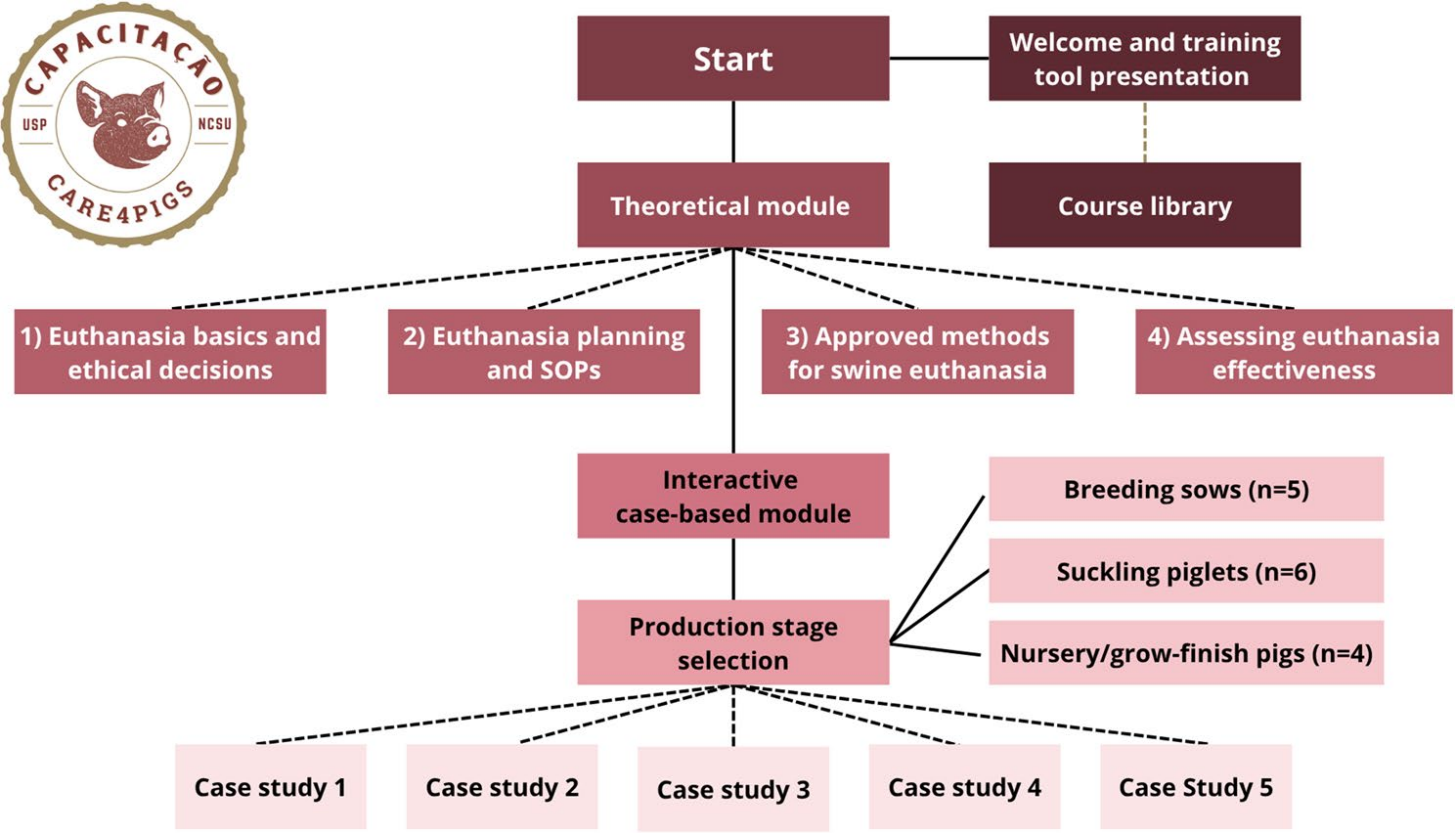
General schematic of the interactive training-tool related to timely on-farm euthanasia. Source: Created by the authors

A schematic overview of the training tool structure is presented in Fig. 1. This visual representation illustrates the pedagogical design and logistical flow of the interactive learning system focused on improving timely and humane on-farm euthanasia decisions.

#### Theoretical module

The content within the theoretical module was based on the “Brazilian On-farm Swine Euthanasia Guidelines” developed by the Ministry of Agriculture and Livestock [4]. It was divided into four video lectures, totaling approximately 40 minutes in length. The lectures were pre-recorded and delivered asynchronously, allowing participants to complete them at their own pace. Each lecture included narrated slides, illustrative images and video footage to support visual learning. The content covered essential concepts including, but not limited to, ethical principles regarding euthanasia, regulatory requirements, methods, indicators confirming insensibility, and confirmation of death. The ethical component emphasized the caretaker’s moral responsibility to prevent unnecessary suffering when recovery is not possible, when treatment is unfeasible within the farm’s resources, or when hesitation delays appropriate action, highlighting accountability and compassion as central values in humane decision-making. The lectures and corresponding topics are summarized in Table 1.

**Table 1:** Summary of the theoretical training module on swine euthanasia, including lecture topics and content covered

Lecture	Title	Description
1	Introduction to euthanasia	Ethical considerations, decision-making criteria, and identification of compromised pigs
2	Planning on-farm euthanasia	Creation of SOPs, selection of appropriate methods by category, and proper restraint techniques
3	Approved euthanasia methods in Brazil	Overview of approved methods, their mechanisms, benefits, limitations, and selection criteria
4	Assessing euthanasia effectiveness	Indicators of unconsciousness, confirmation of death, and mandatory exsanguination as a second step^1^

SOPs: Standard Operating Procedures; ^1^According to Brazilian euthanasia guidelines, the exsanguination is a mandatory second step following the initial euthanasia method, regardless of the technique used [4]

#### Interactive case-based modules

The interactive case-based module consisted of 15 interactive, case-based scenarios illustrating real-life examples of compromised pigs across different production stages, including gestating and lactating sows, pre-weaned, nursery, and grow-finish pigs. These materials were presented in a case-study format, designed to challenge participants to make decisions regarding euthanasia. To ensure content validity and practical relevance, all cases were reviewed by an external advisor and two clinical swine veterinarians with expertise in on-farm animal welfare and euthanasia procedures. Each case scenario was further evaluated to confirm that the decision-making options represented timely or delayed euthanasia according to national and international welfare guidelines [3, 4]. The level of decision complexity and potential risks were classified based on the degree of ambiguity in the animal’s prognosis, ranging from clear-cut conditions, such as severe necrosis or fractures, to borderline situations, such as poor body condition with no evident pain or distress.

The interactive case scenarios were developed for the multimedia training tool over the course of twelve months. The scenarios were designed to simulate common decision-making situations related to swine euthanasia and were distributed across three production stages: breeding sows (*n* = 5), pre-weaned piglets (*n* = 6), and nursery/grow-finish pigs (*n* = 4). The selection of case topics was chosen based on frequency of on-farm occurrences, as reported in scientific literature, international audit data [16] and unpublished industry data. Each case scenario was constructed using photographs and video footage obtained from commercial swine farms, with explicit permission for use in educational settings. A case narrative was created for each scenario, including information such as the pig’s signalment (age, category, production stage), clinical signs, treatment history, and environmental conditions. Participants were prompted to answer the question: *“What would you do next?”* and asked to choose one of several options, including: (1) monitor the animal without intervention, (2) provide treatment and reassess later, or (3) perform euthanasia within a specific timeframe.

**Fig. 2:**
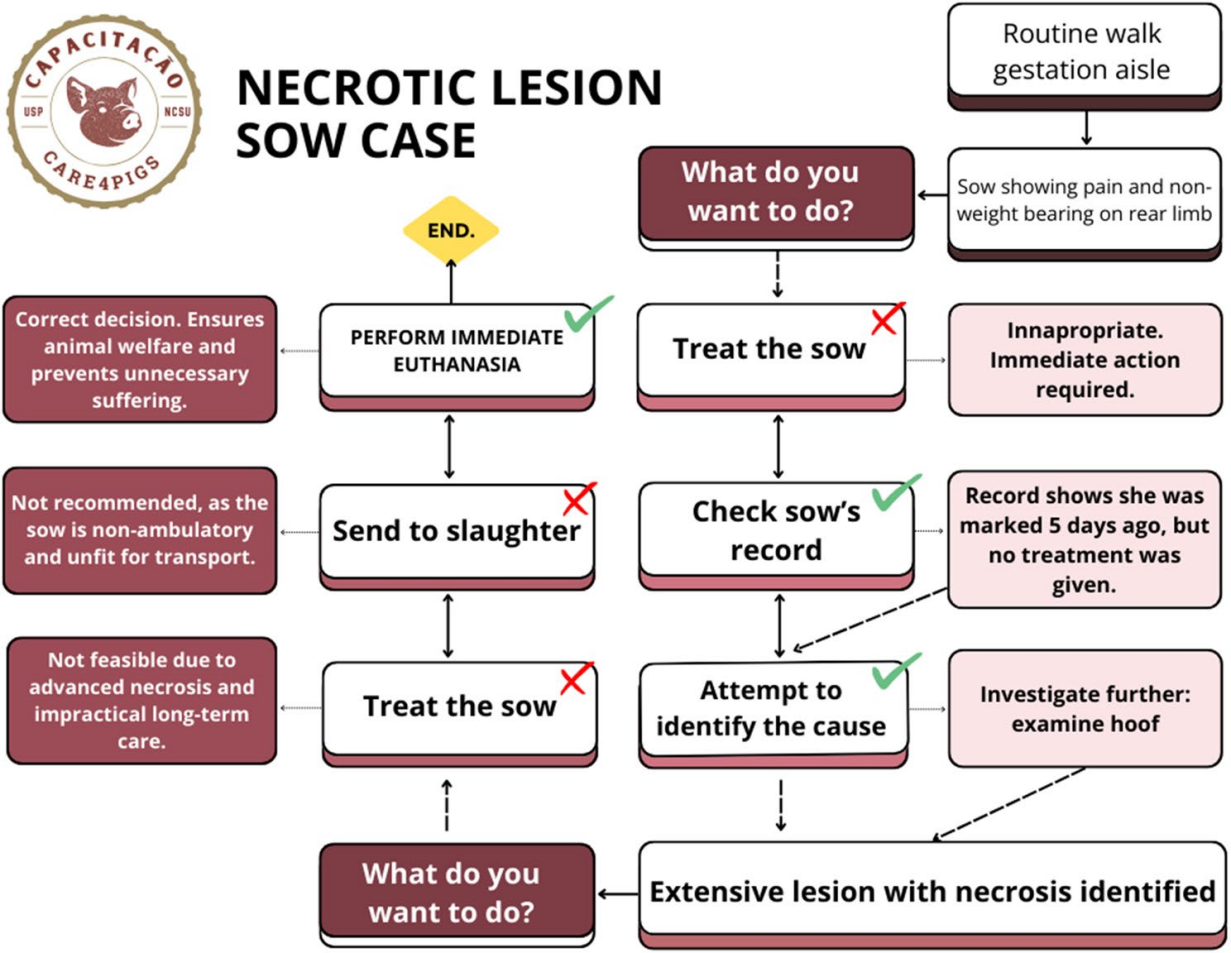
Example of a decision-making structure used in the interactive case-based module. Source: Created by the authors. This scenario involved a sow presenting with a necrotic lesion on the paw. Participants were asked to make sequential decisions, simulating real-world clinical reasoning. Each choice led to different outcomes or feedback, with unsuitable responses prompting students to revisit the decision screen and reflect on alternative actions. This structure was designed to encourage critical thinking and learning through guided reflection. The approach illustrated here was applied across all interactive training cases. In this specific case, the inclusion of “treatment” as an early decision option was intentional, as the animal’s condition was already irreversible and no therapeutic intervention, including pain relief, would have been effective in restoring welfare. This scenario was therefore designed to emphasize timely decision-making and the welfare implications of delayed treatment. The approach illustrated here was applied across all interactive cases

The interactive tool was designed to be accessible on any computer but required internet access. Participants were required to make a decision for each scenario to proceed. When an inappropriate option was selected, feedback was provided explaining the limitations or risks of that choice. Rather than labeling responses as “wrong,” the tool guided learners toward identifying a more suitable option by encouraging them to return to the decision screen and reflect on their choices. This approach was intended to foster critical thinking and support learning through reflection. An example of decision-making structure applied in the interactive case-based module is illustrated in Fig. 2.

Each production stage was presented as an independent module, allowing learners to complete scenarios relevant to their area of interest or experience. The estimated total time to complete the theoretical module and all 15 case scenarios was approximately 2 hours; however, participants can have flexibility to complete only one specific category (e.g., pre-weaned) based on their area of interest. Regardless of the chosen production stage, the theoretical module was required to be completed prior to accessing the interactive case-based scenarios.

### Evaluation of the training tool

The training tool was evaluated across three complementary domains: (1) changes in self-assessed knowledge and confidence before and after training; (2) accuracy of participant decisions withing standardized interactive case-based scenarios, and (3) participant perceptions of usability, realism and overall value of the training tool.

#### Participant recruitment and consent

To evaluate the training tool an extension event was organized at the Swine Research Laboratory (*Laboratório de Pesquisa em Suínos*) of the University of São Paulo, Fernando Costa campus, located in Pirassununga, São Paulo, Brazil. The objective of the event was to provide euthanasia training using the interactive case-based tool previously described. Invitations to participate in the event were distributed through the laboratory’s social media platforms and via email, with the support of the Department of Animal Nutrition and Production.

Students enrolled in veterinary medicine and animal science programs were the targeted audience. To be eligible to participate in the study, students had to be actively enrolled in veterinary or animal science program within Brazil. Participation was voluntary and those interested in participating were required to review and accept an Informed Consent Form provided prior to initiation of the study. Upon providing their consent, participants were required to complete an online pre-training survey to collect baseline data regarding their knowledge and perceptions of on-farm swine euthanasia. This survey is described in detail in the following section.

All participants explicitly authorized the use of their responses and data for research purposes. Students did not receive financial compensation, academic credit or grades for participating in the training. Participation was entirely independent of any coursework or evaluations, minimizing potential bias associated with academic incentives.

#### Pre and post training survey

The survey collected demographic and educational background information, including participants’ age, gender, ethnicity, and the region where they spent most of their upbringing. Academic-related questions included the highest level of education attained, current program or course of study, and year within the program. Participants were also asked whether they had previously taken courses related to swine production or animal welfare and ethics, as well as any prior practical experience with pigs.

Experience with euthanasia was explored through specific questions regarding whether participants had ever performed or assisted in the euthanasia of any animal, which species were involved, and whether they had received any previous euthanasia-related training. Such training could include formal coursework, practical instruction, or theoretical components (e.g., lectures, guest presentations, or seminars). Respondents were considered to have previous euthanasia experience if they had ever performed or assisted in an animal euthanasia procedure before, regardless of whether this occurred before or during their academic training. These survey questions are presented in detail in the Appendix I.

In addition to collecting demographic and background data, participants were asked to self-assess their perceived knowledge and confidence related to euthanasia decision-making in swine, both before and after completing the training program. The same eight items were included in the pre- and post-training surveys to enable paired comparison and evaluation of the impact of the educational tool. These questions focused on key competencies, including the ability to identify euthanasia criteria, recognize clinical signs, make informed decisions, and understand the importance and timeliness of euthanasia.

Participants rated their agreement with each statement using a 5-point Likert Scale: (1) strongly disagree, (2) disagree, (3) neither agree nor disagree, (4) agree, and (5) strongly agree. A “prefer not to answer” option was also available.

The post-training survey included an additional four items specifically designed to evaluate participants’ perceptions of the training tool itself. These questions addressed the realistic nature of the training scenarios, relatability of the material, usefulness in improving euthanasia decision-making skills, and whether the time spent completing the training was worthwhile. Table 2 presents all 12 questions and their corresponding classification as pre- or post-training items.

**Table 2:** List of survey items used in the pre- and post-training assessments

No.	Question^1^	Category
1	I am able to identify when a pig needs to be euthanized.	Pre- and post-training
2	I can recognize clinical signs in a sick or compromised pig.	Pre- and post-training
3	I have sufficient knowledge to handle sick or compromised pigs.	Pre- and post-training
4	I have sufficient knowledge to determine whether a pig should be euthanized.	Pre- and post-training
5	I can identify the health issue in a sick pig.	Pre- and post-training
6	I feel confident in making decisions about euthanasia in pigs when necessary.	Pre- and post-training
7	I understand the importance of performing timely euthanasia	Pre- and post-training
8	I understand the criteria required to make an informed decision about euthanasia in pigs.	Pre- and post-training
9	The training program was realistic.	Post training only
10	The training program presented educational material in a relatable way.	Post-training only
11	The training program met my needs for improving my euthanasia decision-making skills.	Post-training only
12	The time spent completing the training program was worthwhile.	Post-training only

^1^Survey structure adapted from Merenda et al. [15]. Responses were recorded on a 5-point Likert-Scale, from (1) strongly disagree, (2) disagree, (3) neither agree nor disagree, (4) agree, and (5) strongly agree

#### Decision-making activity: interactive case-based application of swine welfare training

The final component of the training consisted of an interactive case-based activity in which participants applied euthanasia decision-making skills to virtual scenarios simulating real on-farm situations. To ensure consistent assessment across participants, all students were instructed to complete the sow module, which included five case studies depicting real-life welfare scenarios involving breeding sows. This standardization allowed for the comparison of responses across a uniform set of scenarios.

Each case was designed to simulate a realistic on-farm situation in which the participant had to determine the most appropriate course of action, including whether euthanasia was indicated. Participant responses were recorded and analyzed to evaluate decision-making accuracy and alignment with established euthanasia guidelines. The decision-making framework and the correct or most appropriate responses for each case are detailed in Appendix II.

### Statistical analysis

Descriptive statistics were used to summarize participant demographics, educational background, and prior euthanasia experience. Categorical variables were expressed as frequencies and percentages, while continuous variables were reported as means and standard deviations (SD) or medians and interquartile ranges (IQR), depending on data distribution.

To evaluate changes in self-assessed knowledge and confidence regarding euthanasia decision-making, Wilcoxon signed-rank tests were applied to paired pre- and post-training responses for each Likert-Scale item. Median values, IQRs, and corresponding p-values were reported. A significance level of *p* < 0.05 was adopted for all inferential tests.

To explore predictors associated with improvement self-assessed scores post-training, univariable and multivariable logistic regression models were built. Score improvement (binary outcome: 1 = improvement, 0 = no improvement) for each Likert-Scale item was modeled as the dependent variable, with demographic and experiential variables (e.g., gender, ethnicity, swine experience, euthanasia training and rural/urban background) as predictors. Variables with *p* < 0.02 in the univariable analysis were considered for inclusion in the multivariable models, and a backwards stepwise approach was taken for model-building. Pearson chi-square was used as a goodness-of-fit test to evaluate model fit. Odds ratio (OR), 95% confidence intervals (CI), and p-values were reported for all models that presented good fit (*p* < 0.05) and at least one statistically significant predictor.

Participant’s performance on the decision-making activity was assessed describing the percentage of correct responses for each scenario component. Frequencies and proportions were calculated per decision point across five interactive sow case scenarios.

All analysis were conducted using Stata 17.0 (StataCorp LLC, College Station, TX, USA).

## Results

### Participant demographics

A total of 47 individuals completed the training and assessments. Descriptive statistics regarding participants’ demographics, educational background, and euthanasia experience are summarized in Table 3.

**Table 3:** Descriptive analysis of demographics, educational background, and euthanasia experience among study participants (*n* = 47)

Variable categorization	Value
*Continuous variables*	*Years*
Age	
Mean	24.45
Standard deviation	4.93
Range	18–36
*Categorical variables*	*% (n)*
Sex, % (n)	
Female	70.21 (33)
Male	29.79 (14)
Racial Identity, % (n)^1^	
White	59.57 (28)
Mixed race	29.79 (14)
Black	6.38 (3)
Asian descent	2.13 (1)
Indigenous	2.13 (1)
Place growing up, % (n)	
Big cities	46.81 (22)
Inner cities	42.55 (20)
Rural	10.64 (5)
Highest degree or level of education, % (n)	
Undergraduate degree in progress	55.32 (26)
Completed undergraduate degree	4.26 (2)
Postgraduate degree in progress	19.15 (9)
Ph.D. in progress	17.02 (8)
Completed Ph.D.	4.26 (2)
Educational background or current course, % (n)	
Veterinary Medicine	38 (80.85)
Animal Science	8 (17.02)
Biological Sciences	1 (2.13)
Current year in program % (n)	
First year	29.79 (14)
Second year	19.15 (9)
Third year	23.40 (11)
Fourth year	6.38 (3)
Fifth year	10.64 (5)
Sixth year	8.51 (4)
Prefer not to answer	2.13 (1)
Completed a swine course, % (n)	
Yes	61.70 (29)
No	38.30 (18)
Completed an animal welfare and ethics course, % (n)	
Yes	68.09 (32)
No	31.91 (15)
Pig experience^2^, %(n)	
Yes	70.21 (33)
No	29.79 (14)
Performed or assisted in the euthanasia of any animal, % (n)	
Yes	61.70 (29)
No	36.17 (17)
Prefer not to answer	2.13 (1)
Euthanasia in several species^3^	
Yes	61.70 (29)
No	38.30 (18)
Received specific euthanasia training, % (n)	
Yes	25.53 (12)
No	74.47 (35)
Performed or assisted in a euthanasia procedure on the last six months, % (n)	
Yes	12.77 (6)
No	85.11 (40)
Prefer not to answer	2.13 (1)

^1^In Brazil, racial identity is self-declared and officially categorized into five groups by the Brazilian Institute of Geography and Statistics (IBGE): *Branco* (White), *Pardo* (Mixed race – a wide range of mixed ethnicities), *Preto* (Black), *Amarelo* (Asian descent), and *Indígena* (Indigenous). The present study uses English equivalents appropriate for international audiences while maintaining alignment with IBGE classifications [17]. ^2^Pig-related experience was classified as follows: internship (46.81%), research (40.43%), work on a pig farm (12.77%), attendance at a technical agricultural high school (2.13%), and work in the swine industry (2.13%). Some respondents reported more than one type of experience. ^3^Animal species in which respondents reported having performed or assisted in euthanasia included: pigs (27.66%), poultry (10.64%), cattle (14.89%), small ruminants (12.77%), horses (6.38%), dogs (21.28%), cats (6.38%), fish (4.26%), rats (2.13%), rabbits (4.26%), turtles (2.13%), and guinea pigs (2.13%). Some respondents reported experience with more than one species

### Multivariable logistic regression analysis

To explore which participant characteristics were associated with greater improvement in post-training scores, logistic regression models were developed for each of the eight Likert-Scale statements (Table 4). No demographic characteristics (as described in Table 3) were associated with improvement in responses to Questions 2, 3, 4 and 5 (Table 2). For questions 1, 6, 7 and 8, specific participant characteristics were associated with increased odds of improvement (*p* < 0.05; Table 4).

**Table 4:** Summary of final mixed-effects logistic regression models identifying participant characteristics associated with increased odds of post-training score improvement (*n* = 47)

Question No.	Statement	Predictor	OR	SE	95% CI for OR	p-value
1	I am able to identify when a pig needs to be euthanized.	Swine course	10.39	1.10	1.21, 89.34	0.033
6	I feel confident in making decisions about euthanasia in pigs when necessary.	Gender (female)	7.95	7.61	1.22, 51.85	0.030
		Prior euthanasia training	4.79	4.49	0.76, 30.04	0.095
7	I understand the importance of performing timely euthanasia.	Ethnicity (white)	11.81	13.18	1.33, 105.18	0.027
		Pig experience (yes)	0.045	0.050	0.005, 0.41	0.006
8	I understand the criteria required to make an informed decision about euthanasia in pigs.	Gender (female)	6.21	5.42	1.12, 34.38	0.037
		Rural residency	0.16	0.16	0.02, 1.09	0.061

OR: Odds ratio. CI: Confidence interval. SE: Standard error. Odds ratios > 1 indicate higher odds of post-training improvement in the respective self-assessed domain. Predictors included in each model were selected based on theoretical relevance and univariate screening

### Training impact on knowledge and confidence

Participants reported improvements in self-assessed knowledge and confidence related to swine euthanasia following training. All eight Likert-scale items on the survey demonstrated increases in post-training scores compared to baseline (*p* < 0.05). Table 5 presents mean (**±**SD) or median (**±**IQR) pre- and post-training results for each statement, including values derived from the Wilcoxon signed rank test.

**Table 5:** Pre- and post-training self-assessment of knowledge and confidence related to swine euthanasia (*n* = 47)

Statement	Pre-training survey		Post-training survey		**ND**^1^	**p-value**^2^
	Mean ± SD	Median (IQR)	Mean ± SD	Median (IRQ)		
(1) I am able to identify when a pig needs to be euthanized.	2.83 ± 1.09	3.0 (2 - 4)	4.11 ± 0.52	5.0 (4 - 5)	+1.28	<0.0001
(2) I can recognize clinical signs in a sick or compromised pig.	3.66 ± 0.92	3.0 (2–3)	4.45 ± 0.58	5.0 (4–5)	+0.79	<0.0001
(3) I have sufficient knowledge to handle sick or compromised pigs.	2.66 ± 1.09	3.0 (2–3)	3.66 ± 0.96	5.0 (4–5)	+1.00	<0.0001
(4) I have sufficient knowledge to determine whether a pig should be euthanized.	2.70 ± 1.08	3.0 (2–3)	3.96 ± 0.75	5.0 (4–5)	+1.26	<0.0001
(5) I can identify the health issue in a sick pig.	2.94 ± 1.09	3.0 (2–3)	3.62 ± 1.03	5.0 (4–5)	+0.68	0.0001
(6) I feel confident in making decisions about euthanasia in pigs when necessary.	2.32 ± 1.16	3.0 (2–3)	3.77 ± 0.84	4.0 (4–5)	+1.45	<0.0001
(7) I understand the importance of performing timely euthanasia	4.55 ± 0.85	3.0 (2–3)	4.91 ± 0.28	5.0 (4–5)	+0.36	0.0043
(8) I understand the criteria required to make an informed decision about euthanasia in pigs.	3.30 ± 1.06	3.0 (2–3)	4.62 ± 0.53	5.0 (4–5)	+1.32	<0.0001

SD: Standard deviation; IQR: Interquartile range; ^1^ND: Mean Numerical difference (Post – Pre); ^2^P-values based on the Wilcoxon signed-rank test

### Participant perceptions of the training tool

After completing the training, participants were asked to rate their perceptions of the multimedia tool across four key dimensions: realistic scenarios, relatability, usefulness for improving euthanasia decision-making skills, and the perceived value of the time invested. Responses were collected using a 5-point Likert-scale, and overall feedback was positive. Table 6 presents the full distribution of responses along with measures of central tendency (mean, median) and variability (standard deviation, interquartile range) for each item.

**Table 6:** Agreement to survey statements (Mean ± SD and Median [IQR]) on a 5-point Likert-scale related to participant feedback on the training tool (*n* = 47)

Statement	5-point Likert-scale evaluation^1^					Mean ± SD	Median (IQR)	% (n) of agreement
	1	2	3	4	5			
The cases felt realistic	0%	0%	6.4%	44.7%	48.9%	4.89±0.31	5.0 (4–5)	93.6% (44)
The cases were relatable	0%	2.1%	6.4%	46.8%	44.7%	4.96±0.20	5.0 (4–5)	93.6% (44)
The tool helped improve my decision-making	0%	2.1%	8.5%	48.9%	40.4%	4.83±0.38	5.0 (4–5)	89.4% (42)
The time spent was worthwhile	0%	0%	6.4%	46.8%	46.8%	4.96±0.20	5.0 (4–5)	91.5% (43)

Participants rated their agreement with each statement using a 5-point Likert-Scale ^1^: (1) strongly disagree, (2) disagree, (3) neither agree nor disagree, (4) agree, and (5) strongly agree. SD: Standard deviation; IQR: Interquartile range

### Decision-making performance on interactive case-based activity

Following the theoretical training, all participants completed five interactive sow case scenarios. The overall average proportion of correct responses across all decision points was 89.1%, suggesting effective knowledge transfer and the ability to apply key decision-making criteria in interactive, real-world-simulated contexts.

Table 7 summarizes the proportion of correct responses for each decision point. While most participants performed well across cases, challenges were evident in more complex or nuanced decision components. For example, only 48.9% chose euthanasia in the presence of a necrotic limb lesion (Case 1), highlighting the difficulty in translating observation into action in ambiguous or borderline cases. In contrast, all participants correctly identified animals requiring further evaluation or immediate intervention in several more straightforward cases.

**Table 7:** Accuracy of decision-making performance across interactive case-based scenarios (*n* = 47)

Case – Case component	Description	% Correct responses
Case 1 – Next step	Painful lesion	100.0 (47)
Case 1 – Euthanasia decision	Necrotic tissue	48.9 (23)
Case 2 – Visual identification	Body condition score 1	97.9 (46)
Case 2 – Euthanasia decision	Based on history	74.5 (30)
Case 3 – Next step	Rectal prolapse	89.4 (42)
Case 3 – Immediate euthanasia	Necrotic rectal prolapse	100.0 (47)
Case 4 – Visual identification	Shoulder sore (contact ulceration)	100.0 (47)
Case 4 – Euthanasia decision	Treatment of the neck wound	89.4 (42)
Case 5 – Euthanasia decision	Gastric ulcer (based on clinical signs)	97.9 (46)

## Discussion

This study developed and evaluated an interactive case-based training tool aimed at improving decision making related to swine euthanasia among veterinary and animal science students, who represent future professionals critical to animal production systems. The findings indicate that the training tool was effective in improving self-assessed knowledge, confidence, and performance within structure decision-making scenarios. In addition, specific participant characteristics were associated with greater improvements across selected outcomes, providing insights into how euthanasia training may be tailored to different learner profiles to support on-farm animal welfare.

Most participating were early-career students ( > 70%, years 1–3), with a mean age of 24 years. Over half were enrolled in veterinary medicine programs, and more than 70% identified as female. Importantly, all racial identities were represented in the study population, reflecting the diversity of students enrolled in Brazilian universities [18]. In terms of prior exposure, the majority had completed coursework in swine production (~62%) and animal welfare and ethics (~68%), and over 70% reported hands-on experience with pigs. The demographic profile of this study is consistent with the broader population of veterinary and animal science programs across Brazil, with younger females constituting the majority of the student body [19, 20].

In this study, 25% of participants had previously received specific euthanasia training, while more than 60% had performed or assisted with euthanasia. These results are in conjunction with findings from the United States [9] and New Zealand [21], where over 50% of the students had been present during or performed euthanasia procedures. Practical exposure has been consistently emphasized in veterinary education as a key component of the learning process that can improve day-one competencies of graduating veterinarians [9, 10]. However, the fact that approximately 40% of participants in the present study lacked any direct euthanasia experience underscores the continued need for structured training opportunities prior to graduation.

When exploring participant characteristic via logistic regression analysis, those who had completed swine specific coursework had higher odds of improving their ability to identify pigs requiring euthanasia (OR = 10.39; *p* = 0.033). These results are aligned with previous research demonstrating that training tools and educational coursework can improve the willingness and ability of those responsible for euthanizing compromised animals to recognize appropriate endpoints [12, 15].

In addition to coursework, gender also influenced outcomes. Female participants had greater odds of improving decision-making confidence (OR = 7.95; *p* = 0.030) and understanding euthanasia criteria (OR = 6.21; *p* = 0.037). Previous research in swine euthanasia has compared gender effects, particularly regarding attitudes and willingness to perform euthanasia. For example, Matthis [22] reported that female caretakers were more likely to choose a less painful method to euthanize a pig, demonstrating empathy toward the animal’s experience. Although our study did not directly measure these constructs, differences in empathy and attitudes toward pig welfare by females may have influenced our results, with female students recognizing the important animal welfare considerations within the euthanasia decision-making. It should be also noted that over 70% of participants were female, and future studies should consider enrolling more males to determine if differences remain when comparing equal population sizes.

Regarding pig-related experience, participants with prior exposure were less likely to show improvement in recognizing the importance of timely euthanasia (OR = 0.045; *p* = 0.006) compared with those without experience. Informal or inconsistent prior experiences may anchor decision-making patterns that are not fully aligned with current best practices, suggesting that participants with prior experience may have already developed their confidence in euthanasia regardless of whether the method or approach was correct. This has been demonstrated in previous studies conducted in Brazil [7, 8] and in North American Countries [23, 24], where caretakers with greater experience often did not improve after training or continued to use inappropriate euthanasia methods despite educational interventions [23, 24]. These results highlight the need for training materials that remain effective across varying levels of prior experience.

Overall, participants demonstrated improvements across all eight self-assessed knowledge and confidence measures following the training. Mean numerical differences were calculated as the change between post-and pre-training scores on a 5-point Likert scale. The most notable gains occurred in decision making confidence (+1.45), the ability to identify pigs requiring euthanasia (+1.28), and understanding the criteria used to inform those decisions (+1.32). These outcomes are consistent with previous interventions using interactive, scenario-based training to enhance recognition of clinical endpoints and confidence in euthanasia-related decisions [14, 15].

In addition, participants’ performance on interactive sow case scenarios further suggests effective knowledge transfer within the context of the training. The overall accuracy across decision points was high (89.1%), however, performance varied depending on case complexity (i.e., the level of ambiguity or uncertainty in assessing prognosis and deciding whether euthanasia was warranted). For instance, only 48.9% correctly chose euthanasia when presented with a necrotic limb lesion, a challenging clinical case in the field. This case demonstrates a broader truth that while foundational knowledge can be taught through standardized tools, euthanasia decisions often involve gray areas requiring contextual judgment, ethical reasoning, and self-awareness [8, 11]. Similarly, the scenario involving a sow with a body condition score (BCS) of 1 was designed to encourage reflection on the threshold between recovery potential and irreversible welfare compromise. In this context, euthanasia would be considered timely only when additional indicators, such as profound weaknesses, inability to stand or access feed, chronic deterioration, or failure to respond to supportive care, indicate that recovery is unlikely. Similar to work by Mullins et al. [12], the ability to apply decision criteria accurately was highest in clear-cut cases, but additional mentorship or discussion may be necessary for more complex scenarios.

The training tool was intentionally designed using visual media, simple language, and realistic case scenarios to optimize learn and retention. Research in adult learning emphasizes that material must be relevant and credible for learners to fully engage [12, 25]. By allowing learners to make mistakes without real-life consequences, the training fostered engagement with ethically complex decisions in a low-risk context. The tool was viewed positively by participants, with over 90% strongly agreeing that the cases were realistic and relatable, and nearly 90% reporting that the training improved their euthanasia decision-making. These results align with previous work in the dairy industry [15], which demonstrated that realistic scenarios and the participant’s ability to relate to the case studies were critical to learner engagement. This tool is now publicly available to any individual interested in learning more about swine euthanasia. Future opportunities to expand this training tool beyond the university and into farms are needed and will likely have a significant and positive impact on pig welfare in Brazil.

These findings also reflect the underlying principles of the Five Domains Model [5], which provides a structured and internationally recognized framework for assessing animal welfare. This model builds upon the traditional Five Freedoms by integrating both physical and mental components of welfare, emphasizing not only the prevention of negative experiences but also the promotion of positive states. Its inclusion in this study highlights the importance of interpreting welfare outcomes holistically, especially in production animals. Within this context, timely euthanasia contributes directly to Domain 3 (Health) by preventing prolonged disease or injury, and to Domain 5 (Mental State) by minimizing suffering, fear, and distress. By training students to recognize compromised states and act appropriately, the tool supports more comprehensive and humane welfare outcomes.

Collectively, the results reinforce the idea that decision-making in animal welfare is not merely a technical skill, but also a cognitive, emotional, and moral process. While structured tools provide an essential educational foundation, they must be embedded in broader educational framework that includes discussion, reflection, and supervised experience. Rather than constituting clinical or predictive validation of euthanasia outcomes, the present study provides evidence of educational effectiveness and learner engagement, addressing a critical gap in Brazilian veterinary and animal science training. By preparing future professionals to approach euthanasia decisions in a more informed, timely, and ethically grounded manner, this tool represents a scalable and practical approach to supporting welfare improvements in Brazilian swine production systems.

## Limitations

Although the results of this study are encouraging, some limitations must be acknowledged. The training tool requires access to computers and a reliable internet connection, which may restrict its use in regions or farms with limited digital infrastructure. While making the tool freely accessible helps reduce economic barriers, technological constraints may still limit its broader adoption in certain production contexts.

During the evaluation of the tool only the sow module of the training tool was used as a representative component to assess learning structure, usability, and decision-making within interactive scenarios. This approach was adopted to standardize the training experience among participants and ensure consistent exposure to content and assessment. Including all production stages would have required substantially more time and could have led to participant fatigue, potentially affecting response quality. Moreover, the tool itself was designed to allow users to select specific modules relevant to their area of work or expertise rather than completing all modules sequentially, which aligns with its intended practical and flexible application.

The evaluation of the training tool was conducted with undergraduate and graduate students, which enabled a controlled and systematic assessment of educational outcomes but may not fully reflect the experiences, challenges, or learning needs of on-farm caretakers and veterinarians. Future studies should extend this evaluation to these target populations, as well as to extension professionals who play key roles in guiding euthanasia practices and monitoring animal welfare in production systems. Finally, as with any educational intervention, assessing long-term retention of knowledge, transfer of learning to daily practice, and measurable improvements in animal welfare outcomes remain a challenge. Although this study demonstrated immediate gains in self-assessed knowledge, confidence, and decision-making performance within training scenarios, longitudinal assessments are needed to determine whether these improvements translate into sustained behavioral changes under field conditions.

## Conclusion

This study demonstrated that an interactive, multimedia training tool can effectively enhance self-assessed knowledge and decision-making confidence related to swine euthanasia among veterinary and animal science students. By integrating realistic, case-based scenarios and fostering critical thinking, the tool supports the application of theoretical concepts within interactive decision-making contexts, representing an important contribution to educational strategies aimed at improving animal welfare practices.

By strengthening the ability to recognize compromised animals (Domain 3: Health) and to act in a timely manner to alleviate suffering (Domain 5: Mental State), the training aligns with the Five Domains Model and its emphasis on holistic, affective-centered welfare. The positive response from participants, together with measurable improvements in confidence, understanding, and decision-making performance withing training scenarios, highlights the potential of this approach as a scalable and evidence-informed educational resource for future professionals involved in swine production systems.

Continued integration of such approaches into academic and industry training programs may foster more rational, timely, and humane euthanasia decisions, benefiting not only animal welfare outcomes but also the well-being of those responsible for carrying them out. Future research should explore long-term knowledge retention, on-farm behavioral changes, and the tool’s effectiveness among farm workers and other non-academic audiences.

## Electronic supplementary material

Below is the link to the electronic supplementary material.

Supplementary Material 1

## Data Availability

The datasets used and analyzed during the current study are available from the corresponding author on reasonable request. The training tool developed is available, for free use at the University of Sao Paulo Extension Platform, access through the link: (https://cursosextensao.usp.br/course/view.php?id=4202).
