# Supplementary material for: Development and evaluation of an interactive case-based training tool for timely on-farm euthanasia decision-making in swine
Source: Porcine Health Manag. 2026 Jan 8;12:2. doi: 10.1186/s40813-025-00483-0 (PMC12857146; doi:10.1186/s40813-025-00483-0)
Supplement: Supplementary file 1 — Supplementary Material 1 [file 40813_2025_483_MOESM1_ESM.docx]

# ***SUPPLEMENTARY MATERIAL***

## **Appendix I – Survey section 1: Demographic and Educational Background of Participants.**

1. **What is your age?** __________________________________________________
2. **What is your gender? (Please select one option)**
   - Male
   - Female
   - Non-binary
   - Other: _______________________________________________________
   - Prefer not to answer
3. **Please specify your racial identity. (Please select one option)**
   - Black
   - Brown
   - White
   - Yellow (e.g., Asian)
   - Indigenous
   - Prefer not to answer
4. **Where did you spend most of your time while growing up? (Please select one option)**

- Big cities
- Inner cities
- Rural
- Prefer not to answer

1. **What is the highest degree or level of education you have received? (Please select one option)**
   - Undergraduate degree in progress
   - Completed undergraduate degree
   - Postgraduate degree in progress (Master’s or Residency)
   - Postgraduate degree (Master’s or Residency)
   - Ph.D. in progress
   - Completed Ph.D.
   - Prefer not to answer
2. **What is your educational background or current course? ______________________**
3. **Which year of your program are you currently in? _____________________**
4. **Have you ever taken a swine production course?**

- Yes
- No
- Prefer not to answer

1. **Have you ever taken a course on animal welfare and ethics?**

- Yes
- No
- Prefer not to answer

1. **Do you have experience with pigs? If yes, please specify. ________________**
2. **Have you ever performed or assisted in the euthanasia of any animal?**

- Yes
- No
- Prefer not to answer

1. **If so, which species? ________________**
2. **Have you ever received specific training on euthanasia?**

- Yes
- No
- Prefer not to answer

1. **Have you performed euthanasia in the last 6 months?**

- Yes
- No
- Prefer not to answer

## **Appendix II – Decision-Making Activity: Practical Application of Swine Welfare Training**

| **Table A.** Case descriptions, questions, and response options for the decision-making activity. | | | |
| --- | --- | --- | --- |
| **Case** | **Description of the case** | **Question** | **Possible choices** |
| ***1*** | Necrotic lesion on the paw – first decision | After identifying this sow, with clear signs of pain, what is the next step? | A) Mark the sow and continue with your daily tasks. **B) Check if there is any information on the sow’s record. C) Try to identify the reason why the sow is showing the problem.** |
|  | Necrotic lesion on the paw – second decision | You have identified the lesion on the sow’s paw, which already has necrotic tissue. What would you like to do now? | A) Treat the sow. B) Separate the sow and send her to the slaughterhouse along with the other cull sows of the week. **C) Perform immediate euthanasia**. |
| ***2*** | Body condition score – first decision | In which of these videos is there a sow with a body condition score 1? | A) Video A (all sows with ideal body condition score)  **B) Video B (four sows with an ideal score and one sow with score 1)** |
|  | Body condition score – second decision | The sow is feeding well and shows no signs of lesions. The sow’s record indicates she had BCS 1 when weaning 15 healthy piglets two days ago. What would you like to do? | **A) Continue monitoring the sow daily and increase her feed level.** B) Treat the sow. C) Euthanize the sow immediately. |
| ***3*** | Rectal prolapse – first decision | After identifying the sow with rectal prolapse, what is the next step? | **A) Euthanize the sow immediately.**  B) Mark the sow and monitor the case daily.  C) Work with the veterinarian and treat the sow. |
|  | Rectal prolapse – second decision | Is it true or false that this sow should be euthanized immediately? | **A) True. The sow should be euthanized immediately.**  B) False. I should inform the Veterinarian and wait. |
| ***4*** | Contact ulceration – first decision | Based on the video, select the female sow you consider needs a more detailed evaluation. | **A) Sow A (visible shoulder sore)** B) Sow B (lying down while the others are standing) C) Sow C (sow is marked) |
|  | Contact ulceration – second decision | The sow has a large lesion on her neck, with tissue around the wound appearing healthy. What should be the next step? | A) Euthanize the sow immediately.  **B) Treat the sow.**  C) Monitor the sow daily to check if the wound becomes infected. |
| ***5*** | Gastric ulcer – only decision | You have identified this compromised sow. What would you like to do? | A) Do not intervene and continue monitoring daily. B) Euthanize immediately. **C) Treat the sow.** |
